# Dataset of surveyed PFAS in water, sediment, and soil of Fountain Creek Watershed, Colorado, USA

**DOI:** 10.1016/j.dib.2023.109280

**Published:** 2023-05-31

**Authors:** Jose Caleb Quezada Davalos, Michael A. Michaud, Luis E. Lowe, Emily N. Hanson, Eric P. Gaulke, Janel E. Owens

**Affiliations:** a Department of Chemistry & Biochemistry, University of Colorado Colorado Springs, 1420 Austin Bluffs Parkway, Colorado Springs, CO 80918, United States; b Fountain-Fort Carson High School, 900 Jimmy Camp Rd, Fountain, CO 80917, United States; c Ultradent Products, Inc., 505 W 10200 S, South Jordan, UT 84095, United States; d Compounder's International Analytical Laboratory, 4760 Castleton Way Suite A, Castle Rock, CO 80109, United States

**Keywords:** PFAS, Per- and polyfluoroalkyl substances, Long- and short-chain PFAS, Watershed, Liquid chromatography tandem mass spectrometry, Contamination

## Abstract

Per- and polyfluoroalkyl substances (PFAS) are widespread and highly persistent organic chemicals with adverse health effects. The US Environmental Protection Agency has issued health advisory limits of 70 ng/L for aqueous concentrations of PFOA + PFOS. In the Colorado Springs, Colorado (USA), metro area, the Widefield Aquifer (groundwater) and Fountain Creek Watershed (surface water) have been contaminated by PFAS from aqueous film-forming foams. Here we present the concentrations of selected linear and branched isomers of legacy PFAS found in surface water (n = 95), soil (n = 83), and sediment (n = 34) samples collected from several creeks of the Fountain Creek Watershed. Collected samples were prepared for high-performance liquid chromatography tandem mass spectrometry (LC/MS/MS) analysis via liquid/liquid extraction and/or solid phase extraction (SPE). This dataset includes the geographic locations of sampled creeks, LC/MS/MS instrumental conditions, method verification data including percent recovery to assess method accuracy and background contamination of PFAS in laboratory reagents and supplies, and determined concentrations of PFAS in water, soil, and sediment samples. These locations were surveyed monthly for a full year and provide a rich dataset to assess influence of sampling location, temporal variability in concentration, and overall contaminant persistence.

Specifications table

**Table:** 

Subject	Analytical chemistry

Specific subject area	Contaminant monitoring in water, soil, and sediment
Type of data	Table
How data were acquired	High-performance liquid chromatography tandem mass spectrometry ***Instruments***: Shimadzu Prominence series LC with in-line mobile phase degasser (DGU-20A5R), LC-20ADXR four-selection pump, thermostat-controlled autosampler (SIL-20AC), column oven (CTO-20AC), and Shimadzu LCMS-8030 triple quadrupole mass spectrometric detector fitted with electrospray ionization operated in negative-ion mode ***Sample preparation products***: Waters Oasis HLB SPE columns (3 cc, 60 mg sorbent bed) ***HPLC column***: Waters XBridge C18, 50 mm × 2.1 mm i.d. × 3.0 µm ***Software***: LabSolutions 5.60 SP2, Shimadzu Corporation
Data format	Analysed Raw
Description of data collection	Environmental samples (water, soil, and sediments) were collected at eight locations in the Fountain Creek Watershed monthly for a full year. These samples were prepared for LC/MS/MS analysis via solid phase extraction (SPE). For soil and sediment samples, liquid-solid extraction steps were performed prior to SPE.
Data source location	Institution: University of Colorado Colorado Springs City/Town/Region: Colorado Springs, Colorado and Fountain, Colorado Country: United States Latitude and longitude for collected samples/data: Location 1: University Village Shopping Center (Costco) 38°54’28”N & 104°49’22”W [Monument Creek] Location 2: Dorchester Park (Dor Park) 38°48’58”N & 104°49’27”W [Fountain Creek] Location 3: Spring Creek Youth Services Center (SCYSC) 38°47’15”N & 104°46’30”W [East Fork Sand Creek] Location 4: Aqua Lane residential street (Aqua Lane) 38°46’15”N & 104°46’14”W [Fountain Creek] Location 5: Bridge at Fountain Creek Regional Trail, mile marker 17 (Bridge) 38°43’44”N & 104°44’00”W [Fountain Creek] Location 6: Fountain Creek Regional Park (FCRP) 38°43’14”N & 104°43’34”W [Fountain Creek] Location 7: Fountain Creek Nature Center – North (FCNC – N) 38°42’35”N & 104°43’01”W [Fountain Creek] Location 8: Fountain Creek Nature Center – South (FCNC – S) 38°40’57”N & 104°42’46”W [Fountain Creek]
Data accessibility	Repository name: Mendeley Data Data identification number: 10.17632/dym93kv36x.3 Direct URL to data: https://data.mendeley.com/datasets/dym93kv36x
Related research article	J. C. Quezada Davalos; M. A. Michaud; L. E. Lowe; E. N. Hanson, J. E. Owens, “Per- and Polyfluoroalkyl Substances (PFASs) in the Fountain Creek Watershed, Colorado Springs, CO, USA: A Yearlong Investigation of PFAS Levels in Water, Soils, and Sediments” *ACS EST Water,***2023**, *3*, 96-105, https://doi.org/10.1021/acsestwater.2c00440

## Value of the Data


 

• These data of selected legacy PFAS in waters, soils, and sediments are crucial to understanding longer-term impact of PFAS release into an urban environment.
• These data of selected legacy PFAS in water, soils, and sediments are of interest to scientific investigators, public health officials, and water utilities seeking to understand the longer-term impact of PFAS release into water resources.
• These data may support further inquiries into additional surface water or groundwater resources or public health investigations to assess impact of PFAS contamination within a community.

## 1 Objective

Per- and polyfluoroalkyl substances (PFAS), which have been used widely in consumer and industrial products, have long-term environmental persistence. In our reported work, we measured PFAS in water, sediment, and soil surveyed at eight locations monthly for a full year within the Fountain Creek Watershed, Colorado Springs, CO, to assess how selected legacy PFAS varied over time and distance [1]. The PFAS concentration data to support that study are included in a Mendeley repository and described here [2]. Beginning in 2016, local news outlets in the Colorado Springs, Colorado, USA metro region reported the contamination of water resources by aqueous film-forming foams (AFFF) [3], [4], [5], [6], which contain PFAS. Several teams of investigators evaluated the impact of AFFF-contaminated water on drinking water utilities and community health impacts [7], [8], [9]. Our contribution here was to report concentrations of PFAS from the Fountain Creek Watershed, which was affected by AFFF-contamination on the Widefield Aquifer. The Widefield Aquifer is a shallow groundwater resource that contributes water to the surface water of the Fountain Creek Watershed. We sampled eight locations within the Watershed for each month over one year to assess the concentrations of selected PFAS in water, sediment, and soil. These data are useful for other investigators seeking to assess long-term impact of PFAS contamination over time and distance.

## 2 Data description

Selected legacy per- and polyfluoroalkyl substances (PFAS), including linear and branched isomers [10], were evaluated in water, sediment, and soil samples collected each month for a full year from eight locations within the Fountain Creek Watershed, Colorado Springs, Colorado (USA). Table 1 includes a description of each sample location, its abbreviated name included in following table data sets, name of the sampled creek, and geographic coordinates. Samples of water (n = 95), soil (n = 83), and sediment (n = 34) were collected at each location each month (Table 2) and were prepared for liquid chromatography tandem mass spectrometry (LC/MS/MS) analyses following sample preparation, which included a solid phase extraction (SPE) clean up. PFAS analytes were separated on a Waters XBridge C18 column and analysed in multiple reaction monitoring (MRM) mode (Table 3). Our method used no internal standard or surrogate standard to account for analyte loss in sample preparation. As such, Table 4 includes determined concentrations via external calibration curves and the pooled recovery data to assess method accuracy for analyte spikes made at 0.400 ng/mL (final concentration after SPE clean up) to all sample types, including water, sediment, and soil. In Table 5, the determined concentrations using external calibration curves for linear and branched PFAS compounds in soils (n = 83) and sediments (n = 34) are reported for each location and each month collected and analysed. In addition to including the determined concentration in the SPE sample extract as ng/mL, the concentration of PFAS in the original soil or sediment is reported as ng PFAS/g soil or sediment (dry weight). Table 6 includes the determined concentrations, by external calibration curves, in replicate analyses of water (n = 95) samples collected from the study locations for linear and branched isomers of selected PFAS. The sample locations, collection date, and creek name are included in this table as well. The determined concentrations, as ng/mL, in the SPE sample extracts are reported along with the original concentration (as ng/L) in the original water sample. Lastly, given the ubiquitous nature of PFAS, the concentrations of PFAS in the LC/MS/MS system (reported as ‘system blanks’) and laboratory reagents used in SPE sample extraction (reported as ‘method blanks’) are included in Table 7. Lastly, Table 8 is a master spreadsheet that includes all analytical standards, laboratory and method blanks, field samples, and spiked samples with a list of where previous concentration data (Tables 4 through 7) may be found. A file folder with all raw data (.lcd) are included in the Mendeley repository. These data files may be opened and manipulated in the LabSolutions software available from Shimadzu Corporation [11].

**Table 1:** Sample location names, abbreviations, surface water, and coordinates.

Location name	Sample name abbreviation	Associated creek	Coordinates
University Village Shopping Center	Costco	Monument Creek	38°54’28”N & 104°49’22”W
Dorchester Park	Dor. Park	Fountain Creek	38°48’58”N & 104°49’27”W
Spring Creek Youth Services Center	SCYSC	East fork, Sand Creek	38°47’15”N & 104°46’30”W
Aqualane residential street	Aqualane	Fountain Creek	38°46’15”N & 104°46’14”W
Bridge at Fountain Creek Regional Trail, mile marker 17	Bridge	Fountain Creek	38°43’44”N & 104°44’00”W
Fountain Creek Regional Park	FCRP	Fountain Creek	38°43’14”N & 104°43’34”W
Fountain Creek Nature Center - North	FCNC - N	Fountain Creek	38°42’35”N & 104°43’01”W
Fountain Creek Nature Center - South	FCNC - S	Fountain Creek	38°40’57”N & 104°42’46”W

**Table 2:** Collection of water (n = 95), soil (n = 83), and sediment (n = 34) samples collected and analyzed from eight locations monthly from the Fountain Creek Watershed.

		Location							
Collection Date		Costco	Dorchester Park	Spring Creek Youth Services Center	Aqualane	Bridge	Fountain Creek Regional Park	Fountain Creek Nature Center - North	Fountain Creek Nature Center - South
9-Jul-18		D	NC	B	D	D	D	D	D
14-Aug-18		B	B	B	B	B	B	B	B
11-Sep-18		B	B	B	B	B	B	B	B
13-Oct-18		A	A	B	A	A	A	A	E
10-Nov-18		B	B	A	B	B	A	A	B
9-Dec-18		A	A	B	B	B	A	A	B
6-Jan-19		B	A	E	D	A	A	B	A
9-Feb-19		B	A	B	B	D	D	A	B
16-Mar-19		A	A	B	B	B	A	A	B
20-Apr-19		B	A	B	B	B	A	A	B
25-May-19		B	A	B	B	B	A	A	B
23-Jun-19		B	A	B	B	B	A	A	B

**A** indicates water, soil, and sediment were all collected and analyzed for the given sampling day.

**B** indicates that water and soil were analyzed for the given sampling day.

**D** indicates that only water was analyzed for the given sampling day.

**E** indicates that only water and sediment were analyzed for a given day.

**NC** indicates not collected.

Note: in many cases, sediment samples were collected but not analyzed owing to limited resources.

**Table 3:** LC/MS/MS Method Parameters.

Analyte	Approximate Retention Time (min)	Acquisition Window (min)	Precursor Ion, m/z	Q1 (V)	Product Ion(s), m/z (ions in bold were used for quantitation)	Q3 (V)	Collision Energy (V)	Dwell Time (ms)
PFBA	0.8	0.1-1.2	212.80	18.0	**169.15**	18.0	10.0	246.0
PFPeA	1.9	1.2-2.6	262.90	13.0	**219.05**	12.0	10.0	122.0
L-PFBS	2.2	1.2-2.6	298.80	12.0	**80.00**, 99.20	12.0, 12.0	30.0, 30.0	59.0
PFHxA	3.1	2.6-3.5	312.90	12.0	**269.00**	15.0	10.0	122.0
PFPeS	3.3	2.6-3.5	348.90	14.0	**80.00**, 99.00	18.0, 18.0	35.0, 35.0	59.0
PFHpA	3.9	3.5-4.1	362.90	11.0	**319.10**, 169.10	10.0, 20.0	16.0, 20.0	59.0
branched -PFHxS	3.9	3.5-4.1	399.00	12.0	**99.05**	18.0	35.0	122.0
PFHxS	4.0	3.5-4.1	399.00	12.0	**99.05**	18.0	35.0	122.0
branched-PFOA	4.4	4.1-7.0	412.90	17.0	**369.05**, 169.10	20.0, 20.0	10.0, 20.0	38.0
PFOA	4.5	4.1-7.0	412.90	17.0	**369.05**, 169.10	20.0, 20.0	10.0, 20.0	38.0
PFHpS	4.6	4.1-7.0	448.90	18.0	**80.00**, 99.05	20.0, 20.0	40.0, 40.0	38.0
branched-PFOS	4.9	4.1-7.0	498.90	12.0	**80.00**, 99.05	12.0, 12.0	45.0, 45.0	38.0
PFOS	5.0	4.1-7.0	498.90	12.0	**80.00**, 99.05	12.0, 12.0	45.0, 45.0	38.0

## 3 Experimental design, materials, and methods

### 3.1 Chemicals and reagents

A certified PFAS stock solution (part no. PFAC-MXC from Wellington Labs, Inc., Ontario, Canada 1.2 mL, 2000 ng/mL concentration with concentrations corrected for the perfluoroalkylsulfonic acids, which were included as the potassium or sodium salts) containing PFAS as follows (with abbreviation) was utilized: perfluoro-n-butanoic acid (PFBA), perfluoro-n-pentanoic acid (PFPeA), perfluoro-n-hexanoic acid (PFHxA), perfluoro-n-heptanoic acid (PFHpA), perfluoro-n-octanoic acid (PFOA), potassium perfluoro-1-butanesulfonate (L-PFBS), sodium perfluoro-1-pentanesulfonate (L-PFPeS), sodium perfluoro-1-hexanesulfonate (L-PFHxS), sodium perfluoro-1-heptane sulfonate (L-PFHpS), and sodium perfluoro-1-octanesulfonate (L-PFOS). This PFAS stock solution did not contain branched isomers of PFHxS, PFOA, or PFOS. As such, concentrations of these isomers in analyzed samples were reported using the linear isomer per EPA guidance [10]. Organic-free HPLC-grade 18 MΩ water produced using a Barnstead E-pure system (Thermo Scientific, Asheville, NC) was used throughout the preparation of analytical standards, sample preparation steps, and for the LC/MS/MS system. Methanol, ammonium hydroxide, and ammonium formate (Fisher Scientific, Fairlawn, NJ) were all LC/MS Optima grade. Ethyl acetate (HPLC-grade) was from Fisher Scientific as were all other chemicals unless specified otherwise.

### 3.2 Sample locations and sample collection protocols

Eight sample locations within the Fountain Creek Watershed were chosen and their names, abbreviations, associated creek, and coordinates are included in Table 1. Table 2 describes the types of samples that were collected at each location at every monthly survey of the field sites. Water and sediment samples were collected in 50 mL FalconⓇ polypropylene centrifuge tubes that had been triple field rinsed. Water samples were stored in a freezer until analysis, at which point they were defrosted prior to SPE. Sediment samples were collected from the soils and gravel samples of the creek bed. These sediment samples were collected from the top 5 to 7 cm of the creek bed in 50 mL FalconⓇ polypropylene centrifuge tubes that had been triple field rinsed with the creek water. Soil samples were collected in 15 mL FalconⓇ polypropylene centrifuge tubes. Soils were collected approximately 6 ft away from the edge of the creek and approximately one foot below the surface to avoid weathering effects or other PFAS contamination by surface features. Sediment and soil samples were prepared for analysis by sifting out large rocks and/or organic matter through a metal screen. Approximately 25 g of sifted soil or sediment were dried in aluminum boats at 80°C for 2 d. The dried samples were transferred to new 15 mL FalconⓇ tubes and stored at ambient temperature (25°C) until prepared for instrumental analysis.

### 3.3 Preparation of analytical standards

An intermediate stock solution of 0.020 ng/µL was prepared by adding 10 µL of the Native PFAS Stock (see *2.1*) to 1 mL of 18 MΩ water. This intermediate stock was stored in a 1.5 mL polypropylene microcentrifuge tube at 4°C and re-made every two weeks.

Calibration standards of 0.04 ng/mL, 0.10 ng/mL, 0.20 ng/mL, 0.39 ng/mL, 0.77 ng/mL, and 1.82 ng/mL were prepared from the intermediate stock solution (0.020 ng/µL) by adding 2, 5, 10, 20, 40, and 100 µL, respectively, to six 1-mL aliquots of 18 MΩ DI water. Standards were vortex mixed. Only Teflon-free Hamilton 700 series syringes (Hamilton Company, Reno, NV) were utilized in the preparation of the intermediate stock and calibration standards. Three hundred µL aliquots of these analytical standards were dispensed into Wheaton 12 × 32 mm polypropylene vials (Fisher Scientific) and capped with a polyethylene olefin snap cap (Agilent Technologies, Santa Clara, CA). Calibration standards were stored for a maximum of two weeks at 4°C.

### 3.4 Instrumental conditions

#### 3.4.1 Preparation of HPLC mobile phases and gradient profile

Mobile phase A was 2.5 mM ammonium formate in organic-free 18 MΩ DDI water modified with 200 µL ammonium hydroxide to pH 8.5 and mobile phase B was 2.5 mM ammonium formate in LC/MS Optima-grade methanol modified with 200 µL ammonium hydroxide to pH 8.5. Mobile phases were prepared as needed and the pH was stable in this buffered system with no impact on chromatographic peak shape. Forty µL injections were made onto a Waters XBridge C18 column (50 × 2.1 mm i.d., 3 µm particle). The gradient conditions were as follows: 35% B at 0 min with a ramp to 95% B at 6.0 min (hold 1.0 min) with a return to 35% B at 7.1 min (hold for 4.9 min) for a total run time of 12 min. The flow rate was held at 0.400 mL/min. Retention times of analyzed PFAS are included in Table 3.

#### 3.4.2 Shimadzu LCMS-8030 operating conditions

The triple quadrupole mass spectrometer was operated using an electrospray ionization source in negative-ion mode at 3.5 kV. Settings were as follows: nebulizing gas at 1.5 mL/min, drying gas at 15 mL/min, desolvation line temperature at 250°C, and heat block temperature at 400°C. Argon was utilized as the collision gas and was maintained at 230 kPa. The mass spectrometer was operated in multiple reaction monitoring (MRM) mode with unit resolution for Q1 and Q3. The instrument was optimized to find the best precursor and product ion(s) for each PFAS along with optimal voltages for Q1, Q3, and collision energy (Table 3). Dwell times for each ion transition were automatically calculated and ranged from 38 to 122 ms. Acquisition windows for closely eluting compounds were established to enhance the sensitivity of the method.

### 3.5 Sample preparation protocols

#### 3.5.1 Water

Collected water samples (Table 6) were prepared for LC/MS/MS analysis using Waters Oasis HLB columns (3 cc, 60 mg sorbent per cartridge, 30 µm particle size; Milford, MA), which were conditioned using one column volume of methanol followed by one column volume of 18 MΩ water. Twenty-five mL of the collected water sample were loaded onto the conditioned column using a volumetric pipette into a 70 mL polypropylene SPE reservoir (Restek, Bellefonte, PA) attached via a Restek SPE cartridge/reservoir adapter. The water sample was eluted from the conditioned column at a rate of 2-3 drops per second. After the entire 25 mL sample had been eluted through the conditioned SPE column, the column was dried under vacuum (10 in Hg) for approximately 10 minutes. PFAS were eluted from the SPE sorbent bed and collected into a 15 mL polypropylene centrifuge tube by adding 1 column volume of methanol (∼ 3 mL) followed by 1 mL ethyl acetate. These solvent eluents containing the analytes were collected into a 15 mL Falcon tube to which three hundred µL of 18 MΩ water were added as a “keeper” solvent, which is a low-volatility solvent added during drying procedures to minimize analyte loss. The methanol/ethyl acetate solvent system was removed by drying the sample to a final volume of ∼400 µL using a gentle stream of nitrogen and mild heat (60°C). The final sample volume was adjusted to exactly 1 mL with the addition of 18 MΩ water by drawing up into a Hamilton syringe and mixing well. A 300 µL aliquot of the sample was transferred to a Wheaton 12 × 32 mm polypropylene autosampler vial and capped with a polyethylene olefin cap to await analysis by LC/MS/MS. According to our QC plan (see *2.6*), field water samples were analyzed in replicate analyses to assess precision (as relative percent difference).

#### 3.5.2 Sediments and soils

One gram of the sieved and dried sediment or soil sample (Table 5), with exact mass recorded on a Mettler XS64 balance, was transferred to a 15 mL FalconⓇ polypropylene centrifuge tube. To each gram of soil/sediment, 3 mL of 75/25 (v/v) methanol/18 MΩ water were added. The tube contents were mixed by shaking prior to sonication in a water bath sonicator for 15 min at 55°C. Following sonication, samples were centrifuged for 5 min at 4000 rpm (or 3005 × *g*) using a Thermo Scientific Sorvall ST16 centrifuge. The supernatant was decanted into a clean 15 mL polypropylene centrifuge tube and saved. Soil/sediment samples were extracted a second time with 3 mL 50/50 methanol/18 MΩ water (v/v) and 15 µL ammonium hydroxide so that the sample pH was ∼9 as measured by pH test strips. Soil/sediment samples were shaken by hand, sonicated for 15 min in a water bath sonicator at 55°C, and centrifuged for 5 min at 4000 rpm (3005 × *g*) as previously described. The supernatant from the second centrifugation was decanted and combined with the first supernatant. The samples were evaporated under under a gentle stream of nitrogen and mild heat (60°C) to ∼2 mL. The remaining ∼2 mL supernatant fraction was transferred to a clean microcentrifuge tube and centrifuged at 17000 × *g* for 20 min using a Thermo Scientific Legend Micro 17 centrifuge. The supernatant was decanted and added to 25 mL 18 MΩ water and cleaned up via SPE as described in *2.5.1*. The methanol/ethyl acetate solution was evaporated to ∼400 µL using a gentle stream of nitrogen and mild heat (60°C). The final sample volume was adjusted to exactly 1 mL with the addition of 18 MΩ water using a Hamilton syringe and mixed well. The 1 mL of reconstituted sample was transferred to a 1.5 mL microcentrifuge tube and centrifuged at 17000 × *g* for 20 min. The supernatant was filtered through a Minisart Regenerated Cellulose (RC) hydrophilic syringe filter (15 mm; Fisher Scientific) and 300 µL transferred to a Wheaton 12 × 32 mm polypropylene autosampler vial and capped with a polyethylene olefin cap. According to our QC plan (see *2.6*), field samples of soils/sediments were analyzed in replicates.

### 3.6 Method verification protocols and quantitation

All samples, including water, sediments, and soils, were analyzed in analytical batches that commenced with analysis of a system blank (see *2.6.1*), followed by six analytical standards (see *2.3*), method blanks, and individual samples. Additionally, quality control (QC) samples included the requisite method blank samples, a field duplicate, and laboratory fortified sample matrix replicate as part of the batch. Calibration check standards were analyzed after every six field samples. Each sample batch concluded with the analysis of at least three analytical standards. All analytical standards were included in the generated calibration curves. If more than three of these values did not meet bias criteria of 100% ± 30%, the entire batch was re-analyzed.

#### 3.6.1 Determination of background PFAS

Laboratory method blanks were included in every batch to assess potential carryover and/or PFAS contamination. The laboratory method blanks were included at the start of the sample extractions. For water samples, laboratory method blanks included 25 mL 18 MΩ water extracted via SPE and prepared for analysis as outlined in 2.5.1. For soils and sediments, laboratory method blanks included the 75/25 (v/v) methanol/water and 50/50 (v/v) methanol/water solutions used for the soil/sediment extractions of 2.5.2. All laboratory blanks were treated as actual samples and carried through the extraction protocols in their entirety prior to LC/MS/MS analysis (Table 7) and are called ‘Method Blanks’. Concentrations of linear and branched isomers are reported in all blanks analyzed.

In addition to these ‘method blanks’ that were processed throughout the entire sample preparation procedure, blank reagent water from the 18 MΩ water system was added to autosampler vials without being cleaned up via SPE. These latter ‘system blanks’ assessed the background level of PFAS within the LC/MS/MS system. These data are also included in Table 7.

#### 3.6.2 Method Verification of Water

To assess accuracy of the SPE protocol, 25 mL laboratory reagent water samples were spiked with 20 µL of the intermediate stock solution (0.020 ng/µL PFAS) and prepared for analysis as described in *2.5.1*. The percent recovery of the spiked PFAS from laboratory reagent water was determined relative to the levels of background PFAS in laboratory method blanks (Table 7).

$$\%Recovery\;of\;PFAS\;from\;Water=100\;\times \;\frac{\left[C_{spiked\;sample}-\;C_{method\;blank}\right]}{0.400\;ng/mL}$$

#### 3.6.3 Method Verification of Soils & Sediments

To determine the accuracy of the extraction protocols, two 1-g soils or sediments per batch were spiked with 20 µL of the intermediate stock solution (0.020 ng/µL PFAS), where a batch included six samples, two spiked samples, and a laboratory blank. These spikes with a theoretical PFAS concentration of 0.400 ng/mL, along with the six samples and laboratory solvent blank, were then extracted using the entire protocol of *2.5.2*. Given that the soil and sediment samples themselves contained measurable levels of PFAS, the spiked samples must be a duplicate of a sample without the spike. The percent recovery of the spiked PFAS were then determined using the equation below:

$$\%Recovery\;of\;PFAS\;from\;Soils=100\;\times \;\frac{\left[C_{spiked\;sample}-\;C_{unspiked\;sample}\right]}{0.400\;ng/mL}$$

To assess the accuracy and precision of the method, the first soil or sediment sample of each batch was analyzed in triplicate (sample 1A, sample 1B, sample 1S) with precision calculated by determining relative percent difference (RPD, %) for samples 1A and 1B, while % recovery (accuracy) was determined for the spiked sample 1S. Additionally, the recovery data for all nine (excluding branched isomers) PFAS were pooled to determine method accuracy for all spiked samples for all matrices throughout the study (Table 4).

#### 3.6.4 Quantitation

The quantitation of the PFAS included in this study were completed for each batch, where every batch included at least *n* = 9 analytical standards with six standards (0.04 ng/mL to 1.82 ng/mL) were included at the start of every batch and three analytical standards (high, medium, and low concentrations) were included at the conclusion of the batch. Calibration check standards were analyzed every six field samples and included in the generated curve for each analyte. All standards were included in external calibration curves, which were fit with a linear regression and 1/*x* weighting. All curves included in this study had *R^2^* values of 0.97 or better. Concentrations of branched isomers were determined using the linear isomer contained with the PFAS stock solution (see *2.1*) [10].

## Ethics statement

The authors have read and followed the ethical requirements for publication in *Data in Brief*. Neither human study participants, animals, nor social media platforms were involved in collecting the data presented here.

## CRediT author statement

**Jose Caleb Quezada Davalos:** Sample extraction and LC/MS analyses (soils and sediments), writing – original draft preparation. **Michael Michaud:** Sample collection, sample extraction and LC/MS analyses (water). **Luis Lowe:** Method development, supervision, reviewing, and editing. **Emily N. Hanson:** Sample extraction and LC/MS analyses (soils and sediments). **Eric P. Gaulke:** Method development for extraction of PFAS from soil samples. **Janel Owens:** Conceptualization, method development, writing – original draft preparation, reviewing, and editing.

## Declaration of Competing Interest

The authors declare that they have no known competing financial interests or personal relationships that have or could be perceived to have influenced the work reported in this article.

## Data Availability

PFAS in Water, Soil, and Sediment of the Fountain Creek Watershed, Colorado Springs, CO, USA (Original data) (Mendeley Data)
